# Bioplatform Fabrication Approaches Affecting Chitosan-Based Interpolymer Complex Properties and Performance as Wound Dressings

**DOI:** 10.3390/molecules25010222

**Published:** 2020-01-06

**Authors:** Hillary Mndlovu, Lisa C. du Toit, Pradeep Kumar, Yahya E. Choonara, Thashree Marimuthu, Pierre P. D. Kondiah, Viness Pillay

**Affiliations:** Wits Advanced Drug Delivery Platform Research Unit, Department of Pharmacy and Pharmacology, Faculty of Health Sciences, School of Therapeutics Sciences, University of the Witwatersrand, Johannesburg, 7 York Road, Parktown 2193, South Africa; 674238@students.wits.ac.za (H.M.); lisa.dutoit@wits.ac.za (L.C.d.T.); pradeep.kumar@wits.ac.za (P.K.); yahya.choonara@wits.ac.za (Y.E.C.); thashree.marimuthu@wits.ac.za (T.M.); pierre.kondiah@wits.ac.za (P.P.D.K.)

**Keywords:** chitosan, polyelectrolytes, biomedical platforms, interpolymer complex, wound dressing, wound healing, IPC fabrication approaches

## Abstract

Chitosan can form interpolymer complexes (IPCs) with anionic polymers to form biomedical platforms (BMPs) for wound dressing/healing applications. This has resulted in its application in various BMPs such as gauze, nano/microparticles, hydrogels, scaffolds, and films. Notably, wound healing has been highlighted as a noteworthy application due to the remarkable physical, chemical, and mechanical properties enabled though the interaction of these polyelectrolytes. The interaction of chitosan and anionic polymers can improve the properties and performance of BMPs. To this end, the approaches employed in fabricating wound dressings was evaluated for their effect on the property–performance factors contributing to BMP suitability in wound dressing. The use of chitosan in wound dressing applications has had much attention due to its compatible biological properties. Recent advancement includes the control of the degree of crosslinking and incorporation of bioactives in an attempt to enhance the physicochemical and physicomechanical properties of wound dressing BMPs. A critical issue with polyelectrolyte-based BMPs is that their effective translation to wound dressing platforms has yet to be realised due to the unmet challenges faced when mimicking the complex and dynamic wound environment. Novel BMPs stemming from the IPCs of chitosan are discussed in this review to offer new insight into the tailoring of physical, chemical, and mechanical properties via fabrication approaches to develop effective wound dressing candidates. These BMPs may pave the way to new therapeutic developments for improved patient outcomes.

## 1. Introduction

Wound healing is an intricate physical systematic series of skin restoration reactions necessitating translational therapeutic approaches capable of aiding the overlapping of inflammatory, migratory, proliferative, and remodelling phases for accelerating tissue filling and restoration. The wound dressing biomedical platforms (BMPs) fabricated from the wound-based translational therapeutic approaches should present an ideal tissue restoration environment by (a) providing and maintaining balanced hydration levels through the absorption and control of the wound exudates; (b) facilitating gaseous exchange across the wound site; (c) preventing secondary infections; (d) controlling and localizing the release of incorporated bioactives; and (e) demonstrating extracellular matrix (ECM)-equivalent physicomechanical characteristics in terms of elasticity and biocompatibility. The ideal tissue restoration environment facilitated by the dressing’s property–performance relationship could easily be detected and followed via observation of the wound healing phases in acute wounds rather than in chronic wounds. This is due to a prolonged or excessive inflammatory phase [1], persistent, continuous infections, drug-resistant microbial biofilms [2,3], and ineffective dermal and/or epidermal cells response to reparative stimuli in chronic wounds [4].

**Figure 1:**
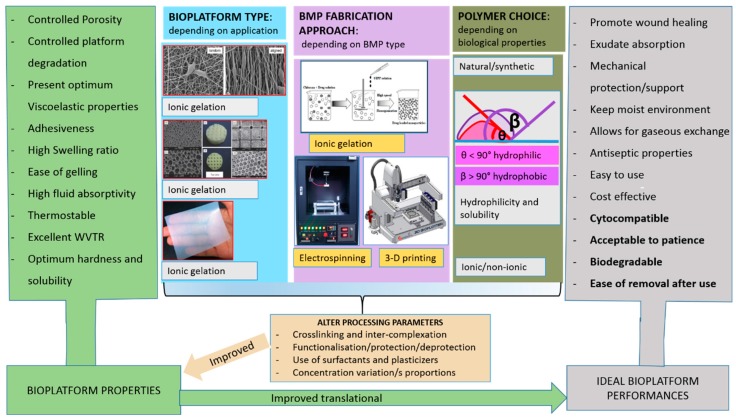
Schematic representation of steps required to design a suitable biomedical platform that meets the ideal requirements for an effective wound dressing. The ideal performances highlighted in bold are amongst the in vitro and in vivo drawbacks of fabricated biomedical platforms (BMPs).

Several property–performance factors should be considered during BMP fabrication to create an optimal tissue restoration environment. These property–performance factors include optimum viscoelasticity, bioadhesiveness, fluid absorption, controlled porosity, and biodegradability (Figure 1). Even if these factors have been taken into account during BMP fabrication, there are still various in vitro and in vivo drawbacks such as BMP side effects, toxicity, low patient compliance, and limited biodegradability as indicated in bold in Figure 1. These drawbacks are a growing concern in BMP fabrication and product costs. Accelerated degradation rates result in the loss of tissue integrity and functions, whereas slow degradation rates can result in mechanical mismatches such as stress shielding, which can impede BMP performance [5]. Therefore, the degradation rate of polymeric matrices must be coordinated with the rate of tissue formation. The performance of chitosan and chitin-based BMPs from in vitro cell studies and in vivo animal studies have displayed effective cell adhesion, biocompatibility, blood clotting control, and wound healing capabilities [6]. To ensure the required performance of the BMP, physicochemical and physicomechanical properties achieved via various approaches of BMP fabrication should be evaluated.

The use of polymers in fabricating BMPs has drawn enormous attention over half a century due to their biocompatibility and ability to be tuned into various BMPs [7]. Although synthetic polymers can easily be modified to facilitate certain reactions and properties, natural polymers present great advantages over synthetic polymers due to their biocompatibility, cytocompatibility, and biodegradability. The biocompatibility character of the BMPs assembled from such polymers allows them to be utilised in the construction of artificial organs and also as drug carriers [8]. The investigation of BMP processing approaches has received much attention in various applications such as fibres [9], beads [10], sponges/scaffolds [11], nanolayered PET films [12], drug-loaded membranes [13], membranes for soft tissue engineering [14], and multilayers [15,16]. However, there is yet to be a BMP engineered that encompasses the ideal biological, physicochemical, and physicomechanical characteristics of the wound environment (Figure 1). The major limitation on recent BMPs is the lack of suitable properties translating to their performance such as effective interaction with biological tissues or cells. Possible considerations that can be taken in fabricating BMPs with tunable properties translating to various performances are shown in Figure 1.

In some BMP applications, the BMP side effects may not be detected in vitro in toxicity studies; instead the BMPs are normally non-toxic and demonstrate effective antimicrobial activity while accelerating wound healing. Skin irritation and rash are among the few side effects experienced for most wound dressings. Limited biodegradability results in frequent dressing changes, and in some cases, the wound dressing needs to be peeled from the healing wound area, resulting in bleeding. These limitations encountered in wound dressings may have originated from the approaches employed in fabricating wound dressing BMPs. Therefore, the current review aims to evaluate the properties affected by the approaches employed in fabricating chitosan-based wound dressing interpolymer complex (IPC) BMPs. In this review, the BMP fabrication approaches were evaluated for the effect on the physical properties (morphology/texture, porosity, water vapour transition rate (WVTR), swelling ratio, solubility, thermal stability, particle size, thermal degradation, and biodegradation), mechanical properties (gelling, interpolymer complexation rate, rigidity, visco-elasticity, tensile strength, and elongation to break point), and performance (cytocompatibility, bioadhesiveness, antibacterial properties, bioactive release, and wound closure). These assessments were proposed to provide insight into crucial considerations when designing BMPs as potential candidates for translation into clinical wound healing applications.

## 2. Polymer Structural Interactions Affecting BMP Property–Performance Factors

The two main classes of polymer/BMP processing approaches that affect the structure and properties of BMPs are physical and chemical crosslinking [8,17]. Chemical crosslinking can be divided into subclasses that include acryloyl group polymerisation, photo-polymerisation, condensation reaction, and co-molecular crosslinking; whilst physical crosslinking includes ionic interactions, hydrogen bonds, and hydrophobic interactions. These types of crosslinking can produce various polymer interactions such as linear homopolymers, linear copolymers, block, random or graft copolymers, polyion–multivalent ions, hydrogen-bonded complexes, and hydrophilic networks [8]. The polymer interactions can be stabilised by hydrophobic domains, physical blends, molecular recognition, self-assembling of polymers, and polypeptides [8]. These interactions can be employed in fabricating several BMPs such as gauze, nano/micro particles, hydrogels, films, fibres, sponges, and printed scaffolds.

**Figure 2:**
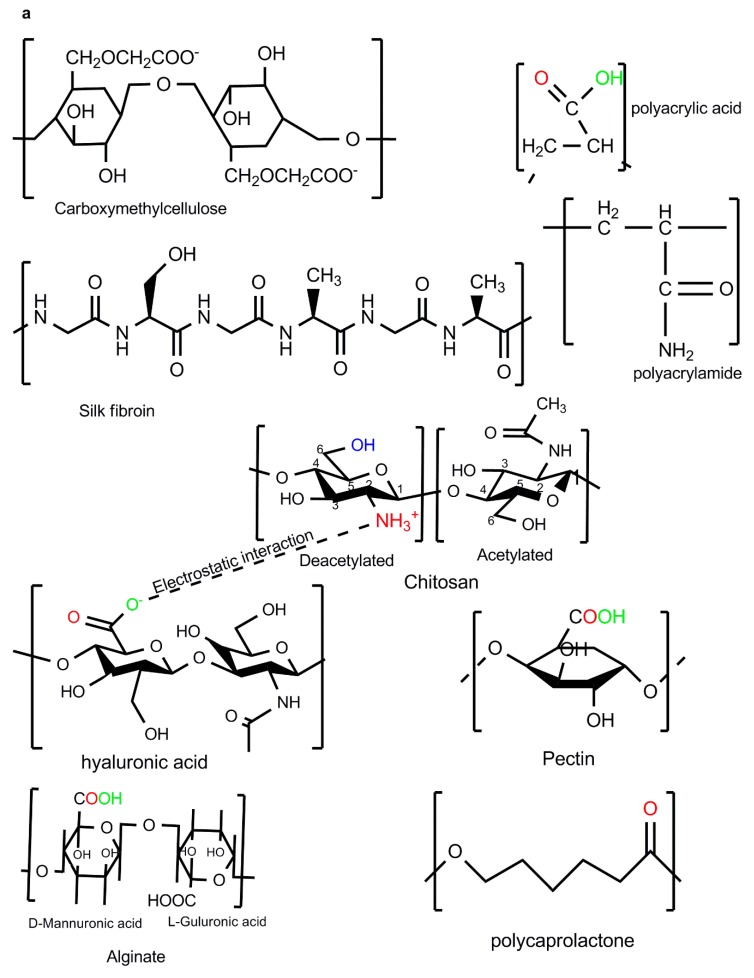

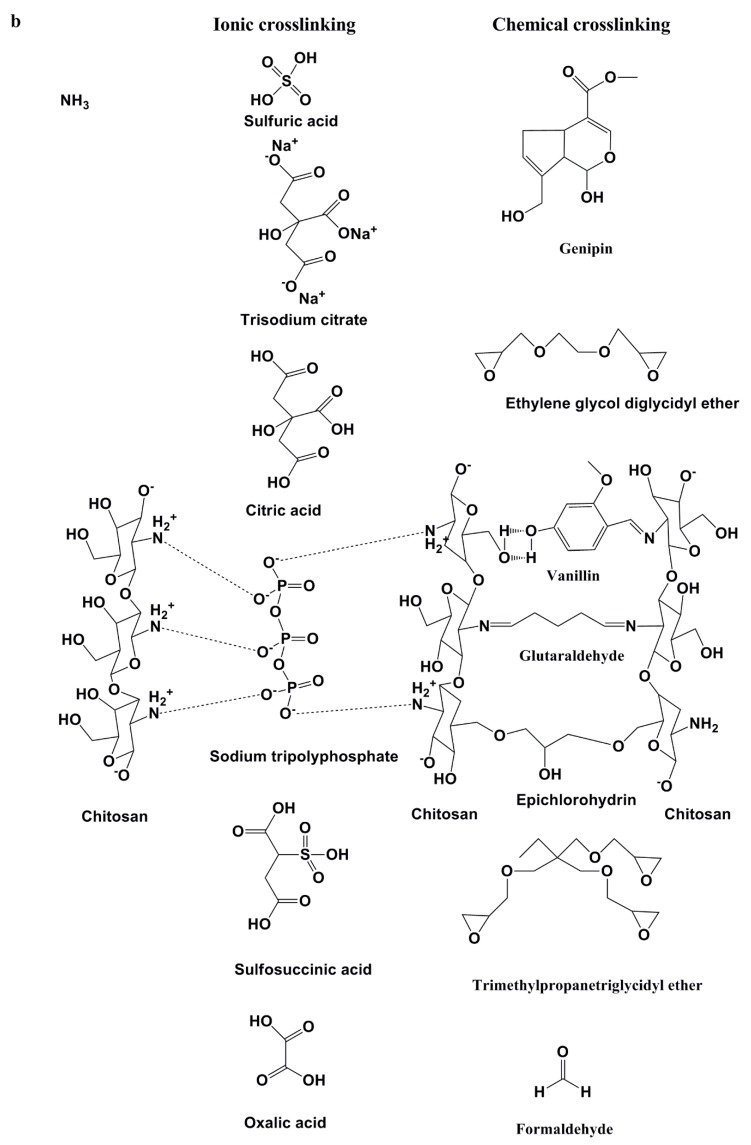
Polyelectrolyte complexations: (**a**) Chitosan-based interpolymer complexes formed with anionic polymers via electrostatic interactions; (**b**) ionic and chemical crosslinkers of chitosan.

Chemical crosslinking provides a permanent junction of networks during the fabrication of BMPs while physical crosslinking forms a transient junction of networks [18]. Detailed information on the biophysical and biochemical synthesis of BMP gels with controlled properties is provided in the literature [19]. Chitosan chemical crosslinkers include genipin, glutaraldehyde, vanillin, formaldehyde, trimethylpropanetriglycidyl ether, ethylene glycoldiglycidyl ether, and epichlorohydrin, whilst physical crosslinking would include sodium tri-polyphosphate, citric acid, trisodium citrate, oxalic acid, sulfosuccinic acid, and sulfuric acid, to name a few (Figure 2b). Chitosan also forms interpolymer complexes with anionic polymers such as alginate, pectin, hyaluronic acid, polyacrylamide, and carboxymethylcellulose (Figure 2a). The following four factors: (a) crosslinking type, (b) synthesis approach, (c) type of BMP, and (d) polymer choice may allow for the tailoring of the BMP property–performance characteristics to meet the ideal requirements (Figure 1). To synthesise BMPs that meet the ideal BMP application requirements, the BMP properties can be targeted and altered until the optimum performance is acquired. The selection of polymers with known optimal properties for the specified BMP application is crucial in the sense that only a few additional properties may be introduced to the BMP during synthesis. To this end, chitosan-based IPC BMPs and their fabrication approaches were evaluated, due to the fact that the properties of chitosan are well established.

The chemical composition of chitosan allows for further processing during crosslinking or interpolymer complexation. Chitosan’s cationic character allows for IPC via ionic interactions with anionic polymers, enables crosslinking with sodium tripolyphosphate or glutaraldehyde, and can easily dissolve in low acetic acid concentrations (Figure 2). The protonation of the NH_2_ group on the C-2 position allows for the solubilisation of chitosan under acidic media (Figure 2) [20]. Chitosan becomes insoluble at neutral pH and the pH of chitosan controls its reactivity/complexation with metals and/or other polymers. The chelating property of chitosan depends on the physical state of chitosan; however, this property depends highly on the NH_2_ content and distribution on each of those physical states [20,21,22]. The chelating property allows for the incorporation of silver and ZnO nanoparticles for improved antibacterial properties with minimal cytotoxicity [21,23]. The interaction of the chitosan NH_2_ group with anionic groups of other polymers allows for the formation of the electrostatic complexes (–COO^− +^NH_3_–) (Figure 2). The electrostatic interaction is crucial for drug encapsulation, controlled release, and the immobilisation of cells. The biomedical properties of chitosan include tissue repair, antimicrobial, immune-enhancing, antioxidant, metal chelating, hypocholesterolemic, lipid binding, anti-inflammatory, anticancer, antitumor effects, and cellular adhesive abilities [23,24]. These biological properties may be affected negatively or positively depending on the type of crosslinkers used (ionic or chemical crosslinking), fabricating approach, and the inclusion of other compounds in the final BMP. Chitosan-based platforms are capable of healing different acute wound types [25]. These polymer properties along with BMP modifications allow for optimum performance on the BMPs.

## 3. Effect of Polymer Processing on Physical and Mechanical Properties of BMPs

**Figure 3:**
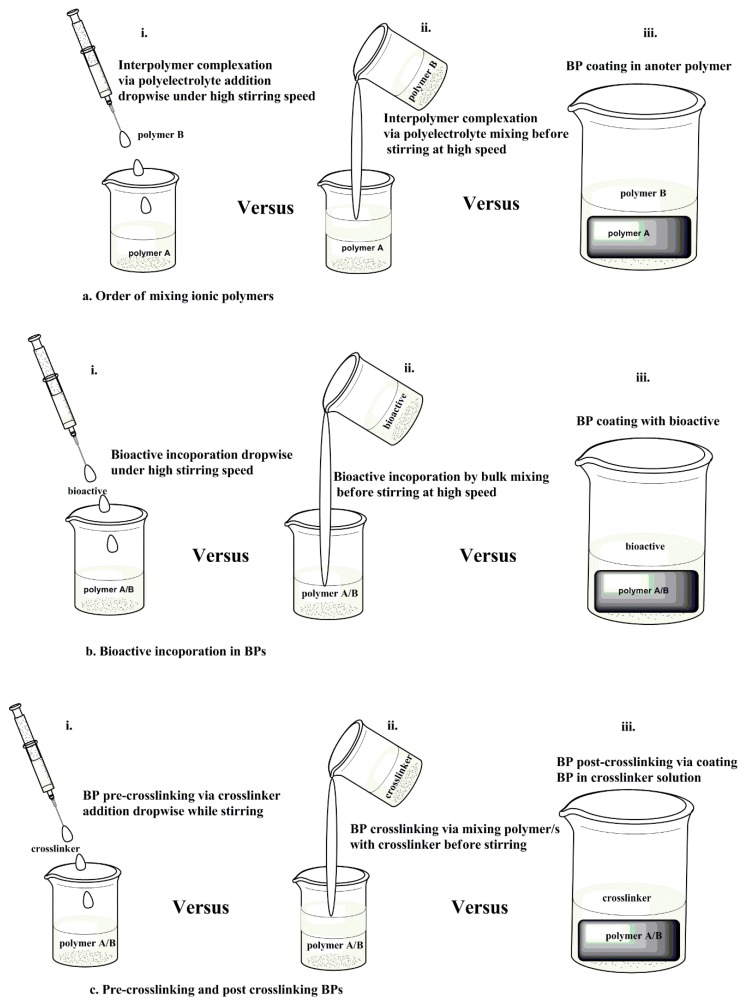
Schematic representation of the processing approaches of ionic polymers. (**a**) Order of mixing polymers; (**b**) bioactive incorporation into the interpolymer complex (IPC), and (**c**) crosslinking of the BMP.

The order of mixing polymers and bioactives during BMP fabrication plays a crucial role in directing the extent of interpolymer complexation, texture, and the strength of the BMP. Chitosan (1% *w*/*v*) and alginate (1% *w*/*v*) were prepared separately and mixed at different proportions followed by the addition of paracetamol to evaluate the effect of polymer ratios on the BMP mechanical strength. The order of mixing that was followed is depicted in Figure 3b(ii). The chitosan sponge displayed more resistance to compression than the alginate or mixed BMP [26]. It was concluded that the order of mixing followed in the study did not produce the columbic interaction between the two materials that had a more robust BMP structure [26]. Chitosan and alginate exhibited greater breaking strengths when they were prepared separately than when they were combined in the BMP [26]. The study indicated that there was no correlation between the hardness and tensile force of the BMPs as chitosan showed high rigidity (hardness) and resistance to breakage (tensile force) while alginate displayed softer texture, even though it had relatively high strength [26]. Furthermore, the BMPs from pristine polymers displayed a regular network while the BMP from the mixture of the two polymers displayed irregular morphology [26]. Changing the processing approach of the BMP as displayed in Figure 3a(i),b(i) could be evaluated by the morphology and mechanical strength of the biomedical platforms.

In another study, chitosan (0.4% *w*/*v*) and alginate (0.4% *w*/*v*) were prepared separately. Chitosan solution was added dropwise to alginate solution at high stirring speed as depicted in Figure 3a(i) followed by dispersion of silver sulfadiazine while stirring. CS/Alg The IPC membranes displayed optimal viscosity and accelerated silver sulfadiazine release at a 1:1 CS:Alg ratio [27]. One of the functions of a dressing is to control evaporative water loss from damaged skin tissue [28]. The evaporative water loss is obtained by calculating the WVTR of the BMPs. The CS/Alg IPC exhibited pH and ionic strength-dependent water uptake properties with the WVTR ranging from 442 to 618 g/m^2^/day [27], this WVTR range falls within the standard range for suitable wound dressings as indicated above in Section 3. The breaking strength of the dry membrane was 52.16 MPa with a wet membrane elongation to break of 46.28% [27]. The elongation to break percentage demonstrates the increase in length at the breaking point of the BMPs with respect to their original length [29]. These properties (elongation to break and breaking strength) provide insights on the elasticity of the BMPs. There was still no correlation between breaking strength and elongation to break of the CS/Alg BMPs as chitosan showed a high breaking strength and resistance to breakage, while the alginate displayed no elongation to break even though it had a relatively high dry breaking strength. The lack of elongation to break in the alginate BMP is due to the point that alginate dissolves in water. Incorporation of silver sulfadiazine did not change the texture of the BMPs and the BMPs displayed regular morphology compared to the irregular IPC formed from different polymeric ratios [27].

Pre- and post-crosslinking of polymers dictates the mechanical strength, matrix hydration, and swelling of the BMP. Chitosan/dextran films were processed followed by the addition of plasticizer (polypropylene glycol) at increasing concentrations and crosslinked (glutaraldehyde) as indicated in Figure 3c(ii) with slight agitation instead of stirring before air drying. The addition of plasticizer did not improve the water vapour penetration on the film, however, the water equilibrium content and swelling increased [30]. Furthermore, the breaking strength and the elongation to break were increased [30]. Chitosan-based BMP was crosslinked with genipin as depicted in Figure 3c(ii). The porosity, swelling capacity, mechanical strength, biodegradation, and antimicrobial properties of the BMP were enhanced by increasing genipin concentration while maintaining moderate WVTR [31]. It is apparent that pre-crosslinking is mostly employed in fabricating BMPs compared to post-crosslinking. This may be due to the fact that the post-crosslinking may be ineffective in altering BMP properties due to the already formed IPC structures, thereby rendering the crosslinkers unable to interact with the polyelectrolytes. Polymer and bioactive mixing/incorporation via the processing approach observed in Figure 3b(iii) is more common than post crosslinking due to the different function of each added biomolecule. The polymer surface coating provides functions such as cell attachment, antimicrobial properties, tissue attachment, and BMP protection from hydrolytic degradation while the bioactive coating on BMPs allows for burst drug release and enhanced antimicrobial properties. Post-crosslinking can also offer properties such as enhanced water uptake and delayed surface degradation of BMP.

## 4. Fabrication Approaches Affecting Physical and Mechanical Properties of Chitosan-Based Interpolymer Complexes

BMP properties are interconnected in the sense that when one property is affected by fabrication approach parameter variations, one or more additional properties will also be simultaneously or sequentially affected by such variations. This has been clearly demonstrated in the literature (i.e., crosslinking of chitosan BMP renders chitosan more insoluble thereby decreasing porosity, fluid uptake, swelling, degradation, and drug release) [32,33,34]. Furthermore, the hydrophilicity and crosslinking density of the crosslinking agents dictate the stability and swelling behaviour of BMPs [32]. Texture and morphology gives information on the hardness, adhesiveness, spreadability, and extrudability of the BMPs. To demonstrate the manner in which texture is affected by these physical and physicochemical properties, a study was conducted on sericin/chitosan-capped silver nanoparticles incorporated hydrogel [35]. The hydrogel properties such as spreadability (work done: 4.3 ± 0.90 mJ), extrudability (239.6 ± 3.93 mJ), hardness (45 to 84 g), and adhesiveness (19 to 39 g) were directly proportional to the concentration (0.5 to 1%) of Carbopol^®^ used in fabricating the hydrogel [35]. The study demonstrated the control of polymer concentration in fabricating aesthetic pharmaceutical products with optimised texture properties.

**Figure 4:**
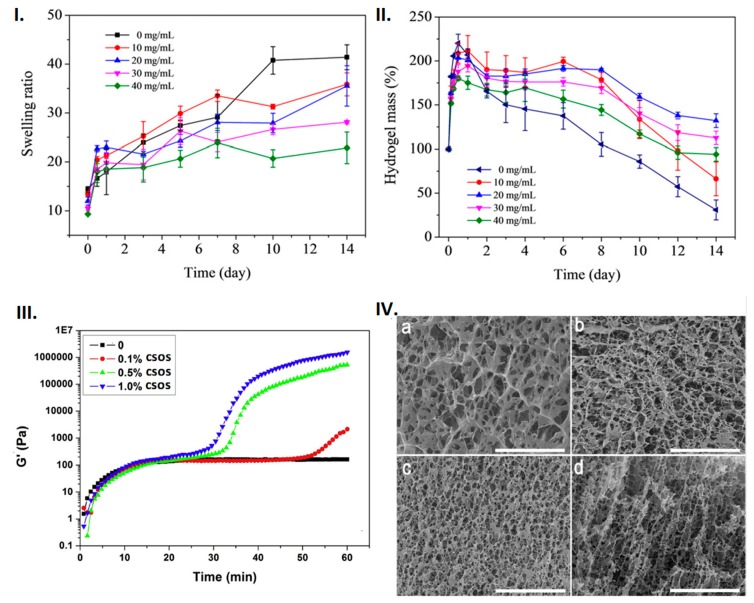
Physical and mechanical properties of the chitosan-based composites: (**I**) Swelling and (**II**) degradation kinetics of tetracycline hydrochloride (TH)/oxidised alginate (OAlg)/carboxymethyl chitosan (CMCS)/gelatine microspheres (GMs) gel with different concentrations of GMs as a function of time in PBS at 37 °C. Image reproduced with permission from Chen et al. [36]. (**III**) Gelling kinetics (of carboxymethy chitosan (CMCS)/alginate/chitosan oligosaccharides (CSOS) hydrogels at different CSOS concentration; (**IV**) Porosity observed in SEM images of CMCS/alginate (1:1) hydrogels with different concentration of CSOS: (**a**) no COS 0.1%, (**b**) CSOS, (**c**) 0.5% CSOS, and (**d**) 1.0% CSOS; (scale bar = 40 μm). Image reproduced with permission from Lv et al. [42].

Porosity, WVTR, fluid absorption, and swelling ratio are some of the physical properties affected by the BMP fabrication and processing parameters. BMPs should have the ability to absorb fluid while maintaining a moist environment on the wound area. Fluid absorption allows the BMP to swell and degrade. When polymeric microspheres with limited fluid absorption capabilities occupy a large space in a hydrogel network, it reduces the amount of fluid uptake by the BMP [36]. A recent study indicated that high concentrations of gelatine microparticles (GMs) result in a decrease in the BMP swelling ratio compared to BMPs without GMs (Figure 4I,II) [36]. This relates to a larger space occupied by GMs in the BMP network structure, thereby limiting the BMP from absorbing large fluid quantities. The swelling and degradation kinetics of chitosan-based BMPs showed that chitosan can swell and degrade more when it is combined with alginate alone than when it is with both alginate and gelatine polymers (Figure 4I,II). This infers that BMP degradation and swelling kinetics can be highly affected by the polymers, drugs, surfactants, and crosslinkers involved in BMP fabrication. Drug incorporation into a BMP may increase/decrease its porosity, thus its fluid uptake and swelling ratio depends on the fabrication approach and processing parameters such polymer proportion and the use of crosslinkers. This has been documented in several studies such as the incorporation of Ag/ZnO into chitosan BMPs, which decreased the porosity from 93% to 81–88% and swelling from 26 times to 21–24 times [37]; incorporation of ibuprofen into PVA/chitosan membranes decreased porosity, however, the membranes were able to maintain high WVTR [38]. Incorporation of ZnO nanoparticles into heparinised polyvinyl alcohol/chitosan hydrogels increased porosity along with the swelling ratio and WVTR [39]. Incorporation of bioactives is crucial for BMP performance, therefore major decisions need to be made in deciding on which property should be prioritised over the other.

Chitosan is insoluble in water, however, processing chitosan with crosslinkers [34], functionalisation to produce *O*-carboxymethyl-*N*,*N*,*N*-trimethyl chitosan (CMTMC) [40], and complexation with other biomolecules such as proteins [41] and anionic polymers can enhance its water solubility. Chitosan solubility is crucial for the activation of the amine groups that participate in the interpolymer complexation reaction with anionic polymers and peptides. The low aqueous solubility of chitosan allows it to be an effective material for absorbing large volumes of water/exudate. Matrix hydration rate, fluid absorption, and swelling ratio are directly proportional to the BMP physical degradation. These three factors are dependent on the BMP type (i.e., sponges and fibres of larger sizes may have low hydration rate, thereby prolonging hydration of the BMP core depending on the processing of the BMP). Partially-crosslinking chitosan beads increased physical degradation by 58% in 14 days with a swelling ratio of 1891.78% in 8 h [34].

Gelling kinetics, rigidity, visco-elasticity, tensile strength, and elongation to break are crucial mechanical properties of BMPs that should tuned towards that of the skin. The mechanical and physical characteristics of wound dressings should facilitate the gaseous exchange by possessing a porous structure while maintaining visco-elastic properties (Figure 4III,IV). The storage modulus allows for the determination of the hydrogel formation kinetics and also for distinctive analyses of stiff or soft hydrogels. A study by Lv et al. [42] employed the ionic gelation approach by stirring varying stock carboxymethyl chitosan (CMCS) and alginate weight ratios (4:1, 2:1, 1.5:1,1:1, 0.67:1, 0.5:1, 0.25:1) and adding D-glucono-δ-lactone (GDL) at different time points [42]. CMCS/alginate/COS hydrogels were produced by the addition of varying chitosan oligosaccharide (COS) concentrations following the above ionic gelation approach [42]. The addition of COS resulted in two-step hydrogel formation kinetics with a more porous morphology (Figure 4III,IV) [42]. The graphs in Figure 4III demonstrate that the combination of chitosan and alginate presented single step hydrogel formation kinetics, whereas a combination of chitosan, alginate, and chitosan oligosaccharides presented two-step hydrogel formation kinetics. Additionally, the hydrogel was stiffer as the chitosan oligosaccharide concentration was increased (Figure 4III,IV). This infers that the chitosan oligosaccharides produce a more rigid hydrogel while maintaining a porous structure. The study provided insights on how chitosan oligosaccharides can be employed in BMP fabrication to control both the viscoelastic properties and morphology of BMPs.

A BMP should present high rigidity (G’) so as to not be easily agitated out of the wound cavity; on the other hand, it should also display elasticity to stretch like normal skin. The tensile strength and elongation to break assessments aid in obtaining the toughness of the material in terms of the force required to stretch the BMP to break point or how much force can be applied to the BMP while still maintaining its elasticity. Investigations on alginate-chitosan hydrogels containing tetracycline-loaded gelatine microspheres indicated an accelerated gelation time with increasing gelation microsphere concentrations [36]. The hydrogels displayed a storage modulus (rigidity) of 9.21 kPa with the BMP being more elastic than viscous [36]. High elasticity to viscosity ratios in BMPs were also observed in other studies [34] as were a storage modulus at the kilopascal range [43], tensile strength at the MPa range [44], and elongation to break between 150% and 300% [44].

**Figure 5:**
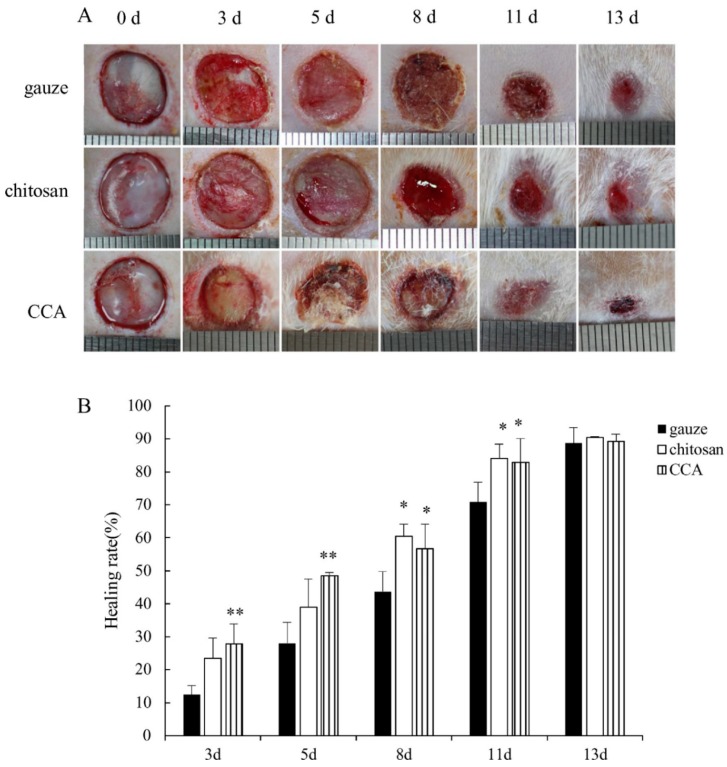
In vivo performance of chitosan-based composites. (**A**) Application of wound dressing platforms with improved properties and performance. The bilayered film/scaffold can be loaded with more than one drug due to the presence of two ionic polymers. The nano/microparticles could also be loaded with more than one drug, and the use of particles would enable the filling of the wound area. (**B**) The wound healing effect of the chitosan-collagen-alginate complex. Wound images were adjusted to the same scale, thereby allowing for a calculation of the wound area. The wound healing rate was calculated by employing the following equation: wound healing rate = (*S*_0_ − *St*)/*S*_0_ × 100%, where *S*_0_ represents the area of the original wound and *S_t_* represents the area of the wound at the testing time (days). Image reproduced with permission from Xie et al. [48].

The BMP performance such as cytocompatibility, bioadhesiveness, antibacterial properties, bioactive release, and wound closure are collectively the target in fabricating biocompatible BMPs. These performances are highly affected by the BMP processing approaches and parameters, which consequently affect the BMP properties above-mentioned. Increasing the bioactive loading in chitosan-based IPCs may reduce cytocompatibility of BMP while enhancing antibacterial, antioxidant, and anti-inflammatory properties [37,39,45,46,47]. A balance between bioactive loading and polymer compositions may be considered in fabricating BMPs with optimal wound healing capabilities. The studies above-mentioned have demonstrated that the property–performance factors are highly affected by the crosslinkers (type and concentrations), polymer combination, drug loading, and processing parameters. The polymeric physicochemical properties and processing parameters such as polymer ratios, order of mixing, solubility, temperature, and pH, and molecular weight affected the property–performance factors more than the type of formulation itself. Chitosan-based BMPs are known to facilitate wound healing, and the processing of chitosan with other polymers or drugs enhances the wound healing state when compared to chitosan-only BMPs (Figure 5) [48]. Chitosan/collagen/alginate complexes displayed enhanced wound healing performance than normal gauze, and chitosan alone (Figure 5).

### 4.1. Scaffold Fabrication Approaches and Their Physical and Physicochemical Properties Impacting BMP Performance

Polymeric scaffolds can be considered as biological substitutes that restore, maintain, and/or improve tissue function [49,50] by delivering cells, drugs, and genes [51]. These biological substitutes can be fabricated into many forms such as a typical 3D porous matrix, nanofibrous matrix, a thermosensitive sol-gel transition hydrogel, and porous microspheres [51]. Scaffolds may be produced via several approaches such as 3-D printing and ionic gelation, which are further discussed in the ensuing sections.

#### 4.1.1. Three-Dimensional Printing of Chitosan-Based IPC BMPs

Three-dimensional printing or bioprinting is one of the recent technologies that offers controlled consistent BMP fabrication via computerised models with high layer-by-layer scaffold design flexibility [52,53]. The advantage of this technique is the fabrication of patient-specific scaffolds with reproducible properties such as orientation and porosity [52]. The drawbacks of 3-D printing are the lack of variation in biomaterial inks that can be processed into self-sustaining BMPs with tunable physical, mechanical, and degradation properties [52]. Other 3-D printing limitations such as bioink printability can be resolved by taking into account the polymeric composition and selecting a specified printing technique such as filaments for fused deposition modelling (FDM), beads (powders) for selective laser sintering (SLS), solutions and gels for direct ink writing (DIW), and solutions for stereolithography (SLA) [52]. The methods for printing polymers is described in depth in the literature [52]; the current article reviews the property–performance effect of various chitosan-based IPCs.

The low mechanical resistance property of chitosan confers a limitation on its printability as an individual material. Mixing chitosan with printable polymers such as pectin or gelatine allows for optimal scaffold printability. To assess the effect of gelatine composition on the mechanical properties and biocompatibility of chitosan/gelatine scaffolds, a gelatine-chitosan polyelectrolyte complex was prepared. It was observed that high concentrations of gelatine (5% and 7.5%) displayed high yield stress and viscosity of the polyelectrolyte complex in a gel-like state (tan α > 1) [54]. It was also observed that low gelatine concentration (2.5%) resulted in poor bioprinting and the sol-gel state (tan α = G′/G″) was below 1, which indicated that the interpolymer complex was at the sol state [54]. The biocompatibility study on the HFF-1 cells indicated that the interpolymer complex with 5% gelatine was more biocompatible than chitosan alone [54].

#### 4.1.2. Ionic Gelation Technique Employed for Fabrication of Chitosan-Based IPC BMPs

Techniques such as electrospinning and 3-D bioprinting offer more consistency in polymer processing and retaining dressing properties. However, these techniques are limited to producing certain dressing types such as nano/micro fibres and/or scaffolds. Alternative approaches do not produce fibres with consistent properties due to variation in the working parameters and elevated degree of errors. That being said, these alternative approaches such as grafting, solvent casting, solution/ionic gelation, and dissolution offer a variety of dressing platforms such as films, nano/microparticles, sponges, scaffolds, foams, and sprays. The ionic gelation technique is one of the most widely employed techniques in the biomedical field due to its non-toxic, organic solvent free, convenient and controllable BMP fabricating process [55,56]. This technique is at the centre of polyelectrolyte (polymer-to-crosslinker/polymer/bioactive) and interpolymer polymer complexes (polymer-to-polymer) where the positively charged primary amino groups of chitosan ionically interact with negatively charged polyions such as sodium tripolyphosphate (TPP), alginate, and hyaluronic acid [56]. This technique includes a number of sub-techniques such as the irradiation and functionalisation of polymers or drugs, which are blended with another aqueous solution. The blending may form particles, sponges, layered hydrogels, or pastes.

The physicomechanical properties of scaffolds may differ depending on the technique employed during BMP fabrication. However, these physicomechanical properties of BMPs should correlate with their application as wound dressings. The composition of various compounds in a blend may affect these physicomechanical properties. To assess the effect of adding organic compounds on the mechanical properties, an oxidised konjacglucomannan (OKGM)/carboxymethyl chitosan (CMCS)/graphene oxide (GO) hydrogel was produced via the Schiff-base reaction between the aldehyde of n (OKGM) and the amino of CMCS [57]. The increase in GO content in the hydrogel increased the tensile strength and modulus of the hydrogel. A fast gelling, high swelling ratio, minimal water evaporation rate, highly porous structure, and stable hydrogel scaffold was produced [57]. The study concluded that the hydrogel scaffold was biocompatible, but lacked data on the rate of degradation, bioadhesiveness, and flexibility of the scaffold. In an independent study, an ice segregation induced self-assembly (ISISA) approach and freeze drying were employed for the fabrication of a glutaraldehyde 3-D chitosan-gelatine scaffold for wound dressing applications. The scaffolds displayed pore sizes of 59 and 75 µm, respectively, which decreased with increasing gelatine proportions [58]. The equilibrium swelling state was reached in 2 h with a maximum swelling ratio of 2450% [58]. The scaffold displayed up to 60% biodegradation in PBS in 21 days, which also increased with increasing gelatine concentration [58]. Physical properties such as swelling, water uptake, and degradation of the scaffolds could be varied for optimal performance. Dry scaffolds could be produced via different drying techniques such as lyophilisation, hot air, and supercritical CO_2_ drying. Buffer solutions could be used to evaluate the swelling properties of the scaffolds. Scaffolds exposed to PBS (pH 7.4) buffer displayed a decrease in the swelling ratio in hot air and super critical CO_2_ drying while not affecting the lyophilised sample [59]. The lyophilisation approach also enabled high drug loading and low drug release when compared to other drying techniques [59]. This is in line with the high surface area observed in lyophilised samples which showed high water uptake thereby exhibiting high drug affinity. The low drug release may be an advantage for prolonged release to maintain the antimicrobial environment. In another study, a solvent gelation coupled with freeze-drying process was undertaken in preparing TiO_2_ loaded collagen/chitosan (Coll-CS) porous hydrogel scaffolds. The increase in nano-TiO_2_ percentage resulted in small pores on the scaffold, thereby causing a rapid increase in the swelling ratio to 925% compared to the dry state [60]. The approach produced a stable scaffold with only 31% degradation in four weeks. The addition of nano-TiO_2_ might have greatly impacted the degradation rate of the scaffold. The poor biodegradability of the scaffold may be useful in chronic wounds and normal deep cuts, burns, and or surface cut wounds. This is due to the requirement that BMPs prepared for normal wounds should possess optimum biodegradability in less than four weeks. Inhibition of *Staphylococcus aureus* and clustered aggregation of red blood cells to stop bleeding was observed on the scaffold, which signified the improved antibacterial properties of the scaffold by adding TiO_2_. The studies above revealed the manner in which different scaffold fabrication approaches can affect the properties and performance of a scaffold. Furthermore, it is also evident that the use of different polymers, metals, and compounds in chitosan blends may have a significant effect on the scaffold degradation and toxicity.

### 4.2. Fibre Physical and Physicochemical Properties Impacting BMP Performance

#### 4.2.1. Electrospun Chitosan-Based IPC BMPs

Electrospinning is a robust, cost effective and consistent nano- to micro-size electrostatic fibre-producing technique that employs electrical force on natural and/or synthetic polymer solutions during fibre synthesis [61,62]. The formed layer of fibres exhibits a porous structure, which allows for gaseous exchange and drainage of the wound exudates while preventing microbial organisms from entering the wound site. The controlled evaporative water loss promoting fluid drainage in blended mats is also controlled by the electrospinning parameters such as polymer concentrations, electric field, and spinning distance [63]. This technique also allows for the incorporation of nano scale drugs that are controllably released in different wound healing stages [64]. Porosity is one of the properties aimed to be controlled by preparing fibres via electrospinning. Although micro scaled fibres may allow microorganisms to pass through the layer, the antibacterial properties of the fibres are proposed to eradicate incoming microorganisms. An alternative approach in dealing with microbial organisms infecting the wound site is to develop a multilayered/bilayered nanofibrous system with the upper layer protecting against external threats and acting as a mechanical structure, while the lower layer can be loaded with the drug to act against inflammation and bacterial growth (Figure 3). To demonstrate the properties of these multi-layered BMPs, a polycaprolactone-hyaluronic acid/chitosan-zein bilayered nanofibrous membrane was developed. This bilayered BMP displayed 90% porosity, 30% degradation in seven days, a Young’s modulus of 4.29 ± 1.46 MPa, and WVTR of 1762.91 ± 187.50 mL/m^2^/day with slow drug release [65]. This system displayed optimum translational performance such as cytocompatibility and cell growth. However, the degradation was low, inferring that the dressing may require changing as the wound heals, which damages the regenerating tissue and thereby limiting patient compliance.

Electrospinning of chitosan (CS) with gelatine (GE) or silk fibroin (SF) has drawn a lot of attention in wound dressing due to its structural stability, biodegradability, and biocompatibility. The CS/SF polymer combination can produce fibres with a high surface to volume ratio, high porosity, and good inter-pore connectivity [66]. Furthermore, the increase in silk fibroin content has previously displayed increased tensile strength and elongation at the breakpoint of the nanofiber membranes [67]. Apart from blending chitosan with other polymers, nanoparticle incorporation into fibres may also affect the fibres’ mechanical properties. To demonstrate the effect of nanoparticles incorporated in fibres, a chitosan (CS)/gelatine (GE) composite nanofiber membrane was electrospun with magnetic Fe_3_O_4_ nanoparticles (NPs) to improve its physicochemical and mechanical properties. The homogeneous Fe_3_O_4_/CS/GE nanofibers displayed well-dispersed Fe_3_O_4_ nanoparticles with hydrogen bonding interaction with the matrix composite, improved thermal stability, and the membrane’s optimal mechanical properties with a 155% augment of Young’s modulus, 128% increase in tensile strength, and 100% boost of toughness from CS/GE [68]. The increase in the concentration of Fe_3_O_4_ nanoparticles from 1% to 4% resulted in a decrease in toughness as well as a decrease in the Young’s modulus and the membrane’s tensile strength [68]. The addition of Fe_3_O_4_ nanoparticles to the CS/GE expanded the trend in the zone of inhibition with chitosan also contributing to the antimicrobial properties of the membrane [68,69,70]. Further gelatine-blended-chitosan (CS/GE) nanofiber mats produced via electrospinning and ultra-sonication displayed increased elastic modulus and elongation to break by using chitosan in the complex [71]. The ultra-sonication produced enlarged porous CS/GE nanofiber mats with improved blood clotting efficiency, cell viability, and cell infiltration when compared with non-sonicated CS/GE nanofiber mats [71]. This provides insights into employing the ultra-sonication step to improve both the physical properties and performance of the electrospun nanofiber mats. Chitosan can decrease the average diameter of the fibre to a constant uniform size, however, the glutaraldehyde crosslinking introduced non-uniformity, and decreased porosity, while increasing the roughness of the nanofiber mats [72,73]. These studies infer that the blending of chitosan with other polymers may improve certain properties of the fibres, but the addition of drugs and crosslinking may also negatively affect these properties. Sodium alginate (SAlg) cannot be electrospun due to its low viscosity. High sodium Alg concentrations (2%) were blended with PVA (16%) to aid the electrospinnability of sodium Alg [45,74]. Silver nanoparticles (AgNPs) were synthesised via the usage of chitosan as a reducing and stabilizing agent, followed by coating the chitosan/AgNP on the electrospun alginate membrane [75]. A smooth surface layer with nanoparticles on the surface, which were due to crosslinking of alginate with calcium chloride, was observed with a high swelling ratio up to 276% and WVTR of 1586–1373 g/m^2^/day with increasing immersion time [75]. However, the coating of alginate nanofibres decreased the WVTR properties. A burst release was observed due to the silver particles situated on the surface of the membrane, thereby exhibiting antimicrobial properties against both Gram-negative and Gram-positive bacteria; however, there was no prolonged drug release [75]. The study above demonstrated that the coating may improve drug release while hampering physical properties such as swelling and WVTR. This indicates that bioplatfrom properties may be tuned towards the application of the BMP. The application of the BMP allows for some properties to be prioritised over others.

#### 4.2.2. Ionic Gelation Technique Employed in Fabrication of Chitosan-Based IPC BMPs

Nanofiber formation and morphology are strongly dependent on the polymer molecular weight, blend ratios, polymer concentration, solvent choice, and the degree of deacetylation of chitosan [76,77]. Chitosan is known to be soluble in acetic acid solution. This highlights that the concentration of acetic acid used in BMP fabrication is capable of affecting the property and performance of BMPs. The effect of acetic acid in CS BMP was evaluated by producing chitin/chitosan-glucan (Ch/CS/GC) nonwoven microfiber mats complexed via a wet-dry-spinning technique, which was aided by the dispersion of the polymers under vigorous stirring [78]. The tensile strength and elongation to break was highly affected by the amount of acetic acid used. The microfiber mats exhibited strong antibacterial properties against Gram-negative bacteria *Escherichia coli*, *Klebsiella pneumonia*, and Gram-positive bacteria *Staphylococcus aureus* with *Bacillus subtilis*. However, enhanced antibacterial activity was observed against the Gram-positive bacteria when the degree of acetylation was increased. The microfiber mats were cytocompatible as there was no cytotoxicity against the mouse fibroblast (NIH-3T3) cell line. Due to the lack of toxicity of the Ch/CS/GC mats, it was suggested that urea/sodium hydroxide aqueous solution can be utilised as a green solvent for dissolution of the Ch/CS/GC complex [78]. There was high cell adhesion to the microfiber mats. Nondiabetic wounds in a rat model treated with nonwoven ChCSGC mats exhibited 95% wound closure in 15 days, which was 23% higher than the cotton gauze employed as a control. The Ch/CS/GC mat-treated diabetic rats displayed enhanced wound closure compared to the untreated diabetic rats.

A simple alteration of the physical treatment of the biomaterial can transform one platform type to another. The effect of the drying approach was evaluated in a CaCl_2_ crosslinked chitosan/alginate polyelectrolyte complex (PEC) to observe the change in the porosity structures of the hydrogel. The gels were prepared by stirring the solution overnight, followed by crosslinking, and thereafter exposed to different drying techniques such as hot air drying at 50 °C, lyophilisation for 48 h, and super critical CO_2_ drying [59]. Hot air drying resulted in compact structures with no macroporosity, which led to the collapse of the PEC network structures. The collapse of the gel structures led to no adsorption due to no or poor porosity [59]. Freezing samples with liquid nitrogen allowed for the frozen water crystals to grow and impede any polymer reorganisation, thereby preventing the collapse of the PEC network structure [59]. This produces networked sheets with interconnected pores as described in the literature for each chitosan and alginate polymer [79,80,81]. Super critical CO_2_ drying produced a 3D nanofibrillated structure without collapsing the PEC network. However, this drying technique required the replacement of water with ethanol, which in turn affected the morphology of the gel and subsequent shrinking and swelling [82]. The swelling ratio increases with increasing surface area or the introduction of porosity. Super critical CO_2_ drying with low surface area displayed the lowest swelling ratio [59]. Collagen/chitosan/alginate (CS/Coll/Alg) fibres were prepared by coating collagen/chitosan on alginate fibres. The optimum water absorption increased swelling to 10 times of its dry weight, had a tensile strength of 0.3638 N/mm ± 0.012 N/mm, and elongation to break of 4.96% ± 0.002 [48]. An optimum cytocompatibility with the cell viability of 100.97% ± 0.071 and higher wound healing ratio was observed in the CS/Coll/Alg composite dressing treated rats than in the gauze or chitosan treated ones [48]. In an unrelated study, a similar concept in fibre preparation was employed; arginine surface-modified chitosan nanofibers were prepared by the attachment of arginine molecules on the surface of chitosan nanofibers using sodium alginate through electrostatic interaction [83]. The nanofibers had an average diameter ranging from 100 to 150 nm, and also showed high viscosity of 1000 cps [83]. The high viscosity property allows for the fibres to stay on the wound cavity instead of moving away in heavily exudated wounds. The fibres did not show any burst release, however, about 85% of arginine was release in 24 h and a wound closure of 93.8% ± 3.1 was observed after nine days [83]. The two studies above demonstrated the use of biocompatible polymers to fabricate biocompatible and cytocompatible BMPs with optimal properties. In these two cases, the biocompatibility of the BMPs were prioritised over the overall BMP properties.

The BMP fabrication approach and properties affecting translational performance in wound dressing applications is summarised in Table 1. The BMP biological performances such as cytocompatibility, antibacterial properties, and wound healing can be controlled by choosing specific biomolecules that can be conjugated with chitosan. The physicomechanical and physicochemical properties of different BMPs depends highly on the fabrication approach and processing parameters such as crosslinking, pH, solvents, and compound compositions. The use of different compounds (polymers, lipids, and proteins), crosslinkers, and the incorporation of bioactives can also affect the property–performance factors of BMPs, as shown in Table 1. The various compounds interacting ionically with chitosan are provided in Table 2.

### 4.3. Gels, Hydrogels, and Membranes Physical and Physicochemical Properties Impacting BMP Performance

#### 4.3.1. Ionic Gelation Technique Employed in the Fabrication of Chitosan-Based IPC BMPs

The polymer crosslinking along with solution viscosity has a major effect on the properties of hydrogels such as tensile strength, bioactive release, and biocompatibility. To demonstrate how crosslinking affects hydrogel viscosity, which consequently affects its property–performance (tensile strength, drug release, cell adhesion and wound healing), a growth factor-loaded chitosan-based hydrogel was prepared. Vascular endothelial growth factor (VEGF) and platelet-derived growth factor-BB (PDGF-BB) loaded visible irradiated light-cured glycol chitosan (GCS) hydrogel systems were prepared. The MPEG/GM-*g*-GC system was prepared by chemically grafting methoxy poly(ethylene glycol) acetic acid (MPEG-COOH) onto (GCS) via a condensation reaction and crosslinking with glycidyl methacrylate (GCM), followed by visible light irradiation for GCS-*g*-GCM amine chain conjugation [86]. The growth factors were incorporated only on the crosslinking reagent at the photo-curing stage, thereby determining the optimum GF concentration for accelerated wound healing. The low viscosity MPEG/GCM-*g*-GCS gel system was observed to have a tensile strength ranging between 56–58 Pa, which is around the range of desired wound healing hydrogels [113]. The low viscosity property of the hydrogel allowed for an in vitro rapid GFs release (60%) within 24 h, followed by sustained release for 30 days. This release behaviour facilitated improved in vivo cell adhesion, spreading, migration, and proliferation associated with accelerated wound healing [86]. The hydrogel samples were compared to the commercially available product Duoderm^®^ and the VEGF/PDGF/MPEG-*g*-GCS hydrogel displayed a faster reduction in wound size. The overall approach used in the study increased the plasticity of the hydrogel and its 3-D porous structures with adequate pore sizes being the key factors contributing to inflammatory and regenerative cell migration and growth. However, the use of radiation light either as a crosslinker or improving polymer properties may have its drawbacks in cytotoxicity. Therefore, the polymers and complexes should be radiated for a very small amount of time. The advantage of increasing the crosslinking concentration is that the pore sizes on the hydrogel decreased greatly. This is crucial for controlled water vapour transmission rate (WVTR). The hydrogels presented optimum cytocompatibility, blood compatibility, and the presence of the crosslinker aided the antibacterial activity of the hydrogel. The hydrogels also exhibited adhesive properties, which were 5 kPa below the commercially available fibrin glue adhesive (Greenplast^®^). This property allowed the hydrogel’s in vivo haemostatic properties to be enhanced by reducing the blood loss from 2025.9 ± 507.9 mg in the control sample to 214.7 ± 65.1 mg in the hydrogel [97]. The hydrogel displayed a comparatively fast rate of wound healing due to the addition of polyaniline.

Gel properties can also be improved via a combination of a few processing parameters together with several coupled techniques. Partially oxidised *Bletilla striatapolysaccharide* (PO-BSP) blended with silver-loaded chitosan particles was achieved by employing ionic gelation in conjugation with the lyophilisation approach to obtain a genipin-crosslinked bilayered film for a wound dressing application [109]. The addition of PO-BSP increased water retention, however, at 0.5% concentration, it decreased the water retention ability of the bilayer films, thereby indicating the closing of pores in the film. Water retention also increased at acidic pH and alkaline conditions when compared to basic pH conditions, which is due to the high porosity and protonation of the free amino group of chitosan that in turn increases the solubility of the polymer chains [109,114]. Genipin crosslinking resulted in the low porosity and low water retention ability of the bilayer films. The tensile strength increased in the PO-BSP blended with silver loaded chitosan and the elongation to break was between 14–19%, however, the increase in the PO-BSP concentration decreased the mechanical properties of the film. The bilayered films displayed optimum L929 cell growth while exerting significant antibacterial activities against *S. aureus* (Gram-positive bacteria), *E. coli*, and *P. aeruginosa* (Gram-negative bacteria) [109]. Optimum wound healing was observed on the bilayered film due to excellent wound exudate absorption, thereby facilitating 88% wound healing by the fourteenth day after application [109]. Although tissue re-epithelisation and cell proliferation were observed on the bilayered dressed wound, there were still several inflammatory cells present, therefore anti-inflammatory agents should be incorporated in the platform in future. Several alginate-chitosan complexes have been fabricated for wound dressing application in the literature [34,48,83]. These two polymers deserve a special review due to their several properties separately and together. Here, a short special case for these two polymers will be reviewed. Sodium alginate readily dissolves in water and its anionic character allows it to interact with cationic polymers and ionic crosslinkers such as CaCl_2_, AlCl_3_, and BaCl_2_ [115]. Alginate’s anionic character allows for the formation of polyelectrolyte complexes with other polymers and facilitates the chelating property with divalent metal ions [116]. The biocompatibility, hydrophilicity, and biodegradability of alginate under normal physiological conditions and its instant gelling character allows it to be employed in various wound dressing applications [11,117]. The use of crosslinkers in alginate BMPs improves the properties of alginate such as maintaining a moist environment, decreasing bacterial infection, facilitating cell attachment, and wound healing [118].

Chemical crosslinking has the ability to improve BMP properties while affecting the BMP cytocompatibility. A droplet extrusion approach was undertaken in preparing tetracycline hydrochloride (TH) loaded gelatine/oxidised alginate (OAlg)/carboxymethyl chitosan (CMCS) hydrogel [36]. In order to demonstrate the effect of chemical crosslinking, the tetracycline hydrochloride (TH) loaded gelatine microspheres were crosslinked with glutaraldehyde while the final hydrogel was crosslinked with oxidised alginate. The use of glutaraldehyde on the TH-loaded gelatine microspheres of 10 to 40 mg/mL concentrations improved the hydrogel’s properties and performance in terms of decreased gelation time and lowered swelling ratios. The storage modulus (G′) and loss modulus (G″) increased along with the microsphere concentration, reaching more than 10 kPa [36]. The cumulative release of tetracycline hydrochloride was lowest for the TH/Gel/OAlg/CMCS hydrogel when compared to the gelatine microspheres [36]. This was indicative of the controlled release by the incorporation of gelatine microspheres into the hydrogel, thereby decreasing the hydrogel’s inhibition zone diameter against *E. coli* and *S. aureus*. However, the sustained release of tetracycline hydrochloride presented effective antibacterial activity against *E. coli* and *S. aureus*.

Calcium chloride ionically crosslinks alginate, thereby forming insoluble alginate. Sodium alginate, chitin, chitosan, and fucoidan were mechanically blended at a ratio of 60:20:2:4 *w*/*w* with a pestle and mortar and allowed to form a paste when exposed to water [107]. The paste was crosslinked with CaCl_2_ and ethylene glycol diglycidyl ether, which formed hydrogel sheets. The alginate/chitin/chitosan/fucoidan fibres displayed high fluid absorption (8 mL in 18 h) compared to a commercial product based on calcium alginate fibres (Kaltostat^®^) [107]. Human dermal fibroblast cells (DFCs) and dermal micro-vascular endothelial cells (DMVECs) did not attach and grow on the alginate/chitin/chitosan/fucoidan sheets; however, the cells grew well adjacent to the fibres. The fibres were elastic and did not deform during wound healing [107]. Furthermore, wound contraction, re-epithelialisation, stimulatory effects on granulation, and capillary formation on day 7 were significant (*p* < 0.05 and *p* < 0.01 for granulation and capillary formation, respectively) on the alginate/chitin/chitosan/fucoidan fibres compared to calcium alginate fibres (Kaltostat^®^) [107]. The study demonstrated the use of calcium chloride as an ionic crosslinker and fluid absorption was enhanced due to the insoluble nature of crosslinked alginate.

Crosslinking can improve the mechanical properties of hydrogels. To demonstrate this effect, N-carboxymethyl chitosan (CMCS) and alginate-based hydrogels were prepared via both electrostatic interaction and divalent chelation with epidermal growth factor (EGF) [108]. CMCS/Alg crosslinked with CaCl_2_ exhibited a G′ value below 30 Pa, indicating a fluid (not a gel) state due to low crosslinking density [108]. The hydrogels displayed a steady increase on the storage modules (G′), loss modulus (G″), and complex viscosity (*η* *) at the initial strain when the crosslinking moieties (either electrostatic or divalent crosslinking) were increased [108]. This is indicative of the higher crosslinking degree with a dual crosslinking approach, which increases the viscosity and elasticity of the hydrogel. Furthermore, the increase in Ca^2+^ moieties decreased the strain of the hydrogel from 60% to 5% [108]. An optimum storage modulus (G’) of about 400 Pa was observed on the CMCS/Alg/EGF hydrogel, which also displayed a 3-D structure with irregular pore sizes (pore diameter of 50–100 µm) [108]. A cell proliferation rate of about 110% on mouse fibroblast (L-929) cells was observed on the hydrogel, and the addition of EGF facilitated the sustained proliferation rate with less than 1% haemolysis ratio [108]. Blood absorption, and the fastest rates of re-epithelialisation and formation of granulation tissues were observed on the hydrogel. The study indicated the dual crosslinking with growth factors and organic compounds, which could be a new trend to be adopted in the wound dressing field.

Interpolymer complexation allows for the formation of a hydrogel without the aid of crosslinkers or surfactants. The CMCS and alginate formed an interpolymer complex hydrogel when exposed in D-glucono-δ-lactone (GDL) [42]. The addition of chitosan oligosaccharides to the hydrogel increased the storage modulus to an equilibrium of 170 Pa, increased proliferation of human umbilical cord mesenchymal stem cells (HUMSCs), increased thickness and integrity of epidermal tissue, increased the formation of collagen fibres, and enhanced the expression of vascular endothelial growth factor [42]. The addition of GDL resulted in a major increase in the storage modulus to 6100 Pa [42], which is too high for wound dressing applications. The high concentrations of chitosan oligosaccharides resulted in cytotoxicity. Moreover, gels do not possess high fluid uptake, which may limit the hydrogel in wounds that have a high exudate volume. A chitosan/alginate membrane was prepared via the ionic gelation approach. The dispersion of alginate was enhanced by the use of surfactants such as Pluronic F68 and Tween 80, followed by crosslinking with CaCl_2_. The effect of the surfactants on the properties of the membrane was reported. The membrane treated with Tween 80 had the highest tensile strength of about 1.5 MPa, elongation at break of 2.1%, fluid uptake from 590 to 1370%, and the membrane increased in thickness up to 3.9 times when immersed in water [106]. The membranes treated with Pluronic F68 had a tensile strength of 1 MPa, elongation at break of 2%, fluid uptake from 774 to 1380%, and displayed increased thickness of about 3.2 times after exposure to water [106]. The tensile strength of both membranes was too high for wound dressing applications; however, the fluid uptake was still in the acceptable range. Although the porosity was not calculated, both membranes were porous, and the membrane treated with Pluronic F68 displayed a higher porosity than the one treated with Tween 80. The membranes treated with either Pluronic F68 or Tween 80 displayed no zone of inhibition against *Pseudomonas aeruginosa* and *Staphylococcus aureus* [106]. This implies that the membranes did not possess any antibacterial properties, therefore an antibacterial drug may be introduced to the membranes.

#### 4.3.2. Polymer Coating, Grafting, Solvent Evaporation, and Solvent Casting Approaches in Fabricating Chitosan-Based IPC BMPs

Polymer processing techniques can be used sequentially or as a substitute for one another. Polymers can be coated with nanoparticles or other polymers. Preparation of biomaterials from such polymers may require both solvent casting and solvent evaporation for improved physicochemical properties. To demonstrate how these physicochemical properties are affected by these techniques, a membrane was prepared via the grafting technique. A microbial transglutaminase (MTGase) biocatalyst was used to graft collagen peptide (CollP) to hydroxypropyl chitosan (HPCS) for wound dressing applications. The degree of substitution on the HPCS/CollP complex affected the moisture-absorption and retention ability, and a mass ratio of 0.34 MTGase:HPCS displayed the optimum moisture-retention ability and cell viability [93]. These two processing approaches displayed improved physical and mechanical properties along with fluctuating BMP performance. In a different study, ibuprofen loaded PVA/CS membranes were prepared via the solution casting method as described in the literature [38,112]. The membranes displayed a large average pore diameter, high porosity, and high swelling ratio (up to 350%) at acidic pH conditions with a far offset WVTR [38]. A remarkable 60% degradation was observed in seven days and no further degradation was observed from day 7 to day 14. Optimum elongation to break of 600% was observed and a low Young’s modulus represented the high elasticity of the membrane at acidic pH conditions, which remained stable for 21 days. The membrane displayed poor mechanical properties at pH 7.4 and basic pH conditions [38]. The drug release studies displayed that the release followed the Fickian diffusion mechanism whereby the solvent transport rate or diffusion is much greater than the process of polymeric chain relaxation [38]. Normal human dermal fibroblast (NHDF) cells adhered and grew in the presence of the membranes and cell viability was not affected by loading ibuprofen into the membranes. Decrease in wound size with no signs of inflammation or the presence of reactive granulomas was observed in wounds treated with ibuprofen-loaded β-cyclodextrins/PVA/CS membranes [38]. This study showed that the addition of drugs on BMPs may improve its properties and performance. However, specific concentrations and pH conditions should be considered.

The use of crosslinkers may also contribute to the soluble/insoluble character of BMPs at certain pH conditions and this can lead to high fluid absorption. A solvent evaporation method was employed in the preparation of [poly (acrylamide (AAm)-co-itaconicacid (IA)] inter-polymer complex (IPC) films to assess the effect of crosslinking and pH on the BMP swelling and fluid uptake. This method allowed for the preparation of an uncrosslinked poly (AAm-co-IA) copolymer, which can be complexed with the CS solution with cationic acid as a crosslinking agent. The presence of these acids increased the solubility of the complex and caused better dissolution, thereby decreasing water absorption. Moreover, the pH conditions allowed for optimum swelling at low and higher pH conditions with water vapour transmission rates (WVTR) ranging between 6000–6645 g/m^2^/day [92]. Although the WVTR were high for the IPC film, the water absorption should be shifted to alkaline conditions by varying the polymer proportions and the amount of crosslinking agent. The pH dependent swelling ratio allows for the film to be optimised to best suit the wound environment type. These approaches demonstrate the use of other polymers and crosslinkers as potential chitosan complexes for improved biomaterial properties.

### 4.4. Sponges Physical and Physicochemical Properties Impacting BMP Performance

#### 4.4.1. Ionic Gelation Technique Employed in Fabrication of Chitosan-Based IPC BMPs

Chitosan can form an IPC with collagen, which can be further crosslinked with glutaraldehyde, epichlorohydrin, and tannin acid. Coll/CS blend sponges were crosslinked with tannin acid and freeze-dried. A uniform texture with abundant pores sizes ranging between 145–240 µm was observed on the sponges, which displayed 60 times more swelling than the dry ones [91]. However, the hydrophilicity of the sponges resulted in them forming a gel when immersed in water. The sponges were not thermally stable, and showed low tensile strength and elongation to break [91]. The Coll/CS sponges displayed improved antibacterial properties due to the rough surface property of the sponge, which prevented bacterial replication, and the presence of tannic acid and chitosan aided this antibacterial property. The histological analyses of the Coll/CTS sponges displayed improved wound healing in 12 days with minimal cytotoxicity. The antimicrobial property of chitosan-based sponges was also improved by adding silver. AgNP-loaded chitosan-l-glutamic acid (CSG)/hyaluronic acid (HA) IPC sponges were prepared to evaluate silver’s effect on the properties of the sponges. The addition of silver on the sponges changed the porous structures’ smooth surface and increased the pore sizes on the sponge to 150–250 µm and folded structures. A tensile strength of 0.04 MPa and elongation to break of 300% was observed, which suggested resistance to deformation properties [104]. The sponges displayed a high swelling ratio of 2900% due to their high porous structure, however, the addition of silver nanoparticles decreased the swelling ratio [104]. CSG without AgNPs displayed no antibacterial properties while the addition of AgNPs at increasing concentrations increased the inhibition zones against *E. coli* and *S. aureus* [104]. The sponges were also cytocompatible as they displayed 80% L929 cell viability in 24 h, indicating low toxicity [104]. However, the cell viability may have decreased as more silver was released by the sponge. The in vivo studies displayed ulceration and oedema with 5% contraction in wounds treated with the gauze (control) in three days whereas the wounds treated with the sponges exhibited wound healing with calluses, slight inflammation, and up to 69% wound contraction at day 3, with crusting observed in 11 days [104]. In a different study, chitosan/silver sulfadiazine (CS/AgSD) composite sponges were prepared by the dissolution technique and freeze drying. The 80% porous three-dimensional network structures were observed on the sponges with a 2% porosity decrease due to AgSD loading [103]. The swelling of 3980% was observed on the sponges after 4 h and also decreased with the incorporation of AgSD [103]. The addition of silver to the sponges exhibited antibacterial activity against *E. coli ATCC 25922, C. albicans CMCC(F) 98001, S. aureus ATCC 6538*, and *B. subtilis ATCC 9372* while maintaining more than 84% of HEK293 cell viability [103]. In another study, gamma irradiation crosslinking was employed in fabricating chitosan (CS)/gelatine (Gel)/polyvinyl alcohol (PVA) hydrogels to assess its effect on the BMP properties. The tensile strength, elongation to break, and gel content decreased with increasing the CS/Gel ratio and a maximum of 85% gel content was reached at an irradiation dose of 40 KGy [44]. A 3-D microporous structure of the hydrogel allowed for a small water evaporation rate with saturated swelling reached in 24 h [44]. A low blood clotting index was observed, which enabled optimum coagulation. This hydrogel is useful in bleeding wounds; however, it can be challenged by wounds with a larger volume of exudates, which may disturb the porous network of the sponge.

Chitosan and alginate are natural polymers that are widely used for interpolymer complexation to produce optimum BMP performance such as cell adhesion, spreading, and wound healing [119]. To demonstrate the performance of such biomaterials, collagen (Coll) was sequentially complexed with both natural crosslinkers chitosan (CS) and alginate dialdehyde (ADA) for thermostability and antibacterial properties. A ten-fold water uptake was observed on the sponges, which was crucial for blood clotting and wound healing. A higher denaturation temperature (Td) and 70% antibacterial activity associated with the higher degree of crosslinking was observed on the complex due to addition of ADA as a crosslinking agent [90].

#### 4.4.2. Phase Separation and Grafting Approach for the Fabrication of Chitosan-Based IPC BMPs

A modified thermally-induced phase separation approach was undertaken in the fabrication of absorbable/non-absorbable levofloxacin-loaded chitosan/2-hydroxyethylacrylate (CS-g-PHEA) sponges as a topical wound delivery dressing. The sponges displayed an average pore size diameter ranging between 152 µm and 225 µm, which greatly decreased with levofloxacin loading. Sixty percent of the PHEA was grafted into chitosan, rendering the sponge partially soluble in alkaline conditions with a saturated swelling ratio of 750% in 4 h, which demonstrated a major weight loss after 4 h [99]. The sponge exhibited 80% degradation on the first day, which was due to the degradation of chitosan by lysozyme. The low tensile strength indicated the poor stability of the sponge, which was improved by the addition of PHEA, thereby rendering the sponge more flexible. A complete levofloxacin loading was achieved in 6 h whilst it was rapidly released in the first hours with a 90% burst release, thereby inhibiting bacterial (*P. aeruginosa and S. aureus*) growth with unquantified L929-fibroblast cell viability after 24 h [99]. Although the sponge demonstrated antibacterial activity, it may be toxic in the long run and the study only presented antibacterial activity and cytotoxicity for only 24 h, which is not long enough to make a conclusive decision about the sponge performance as a potential wound dressing. 

## 5. Standard BMP Properties for Wound Dressing Applications

The properties of the BMP should be close to those of human skin. Most BMPs possess high strengths compared to the skin, which impairs cellular interactions with the BMP. The tensile strength of BMPs should be at the Pascal range, rather than a kilo or mega Pascal range. When the BMP presents extended elongation to break, it implies that the BMP is elastic, which is crucial for skin regeneration. The above-mentioned studies demonstrated that the tensile strength should be around 56–58 Pa. The swelling ratio could be varied to best fit certain wound types. However, a swelling rate of 100–900% is acceptable. The BMP hardness, porosity, and solubility can be varied to suit specific dressings and interactions. The WVTR for normal skin is 204 g/m^2^/day and for wounded skin from 279 to 5138 g/m^2^/day, depending on the type and nature of the wound. Therefore, the BMP WVTR proposed should be 250–2500 g/m^2^/day. All the BMP types such as scaffold/sponge/fibre/membranes, gels, nanoparticles, microparticles, beads/macromatrices, and gauze should adhere to these properties in order to facilitate optimum interaction with the wound environment.

## 6. Conclusions

BMP design considerations for improving the property–performance factors should be taken into consideration during BMP fabrication. The studies reviewed demonstrate that BMP properties and their translational performance are highly affected by the BMP type, polymer, and additives, conjugation choices, fabrication approach, and processing parameters. All these factors are interconnected in producing biomaterials with the optimum properties and performances. Different biomedical conditions require different biomedical platforms for treatment. A drug delivery platform may require a steady release for chronic treatments and a burst release for pain relieving applications. This implies that the platform type dictates the release profile, which also depends on the fabrication approach.

The fabrication approach may yield BMPs with drugs situated on the surface, or in the case of particles, the drug would be incorporated inside the particles. The type of interaction between drugs, crosslinkers, and polymers may also affect the application of the BMPs. In cases where there is strong interaction (covalent interaction) between the drug molecules and polymers, the drug may release slowly or require other molecules that the drug presents a high affinity for. The processing parameters such as concentration, temperature, pH, conductivity, type of crosslinkers, and quantities highly affect the properties of biomaterials. As described in the above studies, the use of different crosslinkers and concentrations may increase or decrease the porosity, polymer interactions, BMP strength, and subsequently BMP performance such as degradation, drug release, and wound healing. It is advantageous to conjugate polymers with different properties that further improve the properties and performance of the BMP. However, the interaction of the polymers should not emanate in the release of toxic derivatives after degradation. The selection of additives such as growth factors, antibiotics, plasticizers, metal ions, and crosslinkers should solely be to improve the properties and performance of the BMPs with minimum in vitro or in vivo toxicity. Various platforms can be optimally produced by a selected number of polymers; therefore, it is crucial to select the best polymers for a specific platform. However, modification of polymers via crosslinking, ionisation and/or functionalisation can render many polymers fit for the production of many BMPs. The studies reviewed demonstrate that modifications of the polymer or complex may decrease other properties and performances such as porosity, swelling, gelation, adhesiveness, and degradation. Therefore, a degree of polymer/complex modification may be applied to improve those properties.

## Figures and Tables

**Table 1:** BMP fabrication approach and properties affecting translational performance in wound dressing applications.

Polymer Composite and Approach	BMP System	Platform Properties	Platform Performance	Ref.
Dissolution of CS/Alg crosslinked with TTP and CaCl_2_	Gels	Constant pore size, thermal stability, rheology, chemical stability	Biodegradation, cell proliferation, antibacterial activity	[84]
Mixing glycerol and molecularly imprinted polymer solutions	Gel	Drug release controlled by diffusion and swelling	Antibacterial activity but no significant data for improved wound healing	[84]
Ionic gelation and irradiation	Gel	Decreased crosslinking increased water uptake and platform elasticity	Inhibits Gram-positive bacteria and does not inhibits Gram-negative bacteria	[85]
Polymer coating and irradiation	Hydrogel	Increased elasticity, maintained 3-D porosity	Burst and sustained release, accelerated wound healing	[86]
Polymer coating	Membrane	Increased tensile strength and reduced porosity	Antibacterial, accelerated wound healing with minimum scar formation	[87]
Polymer coating	Scaffold	Increased solubility and water uptake	Antimicrobial and wound healing	[88]
Polymer casting	Film	transparent, soft, flexible	increased cell proliferation	[89]
Electrospinning and ultra-sonication	Scaffold	Porous, decreased tensile strength	blood clotting efficiency, cell viability and cell infiltration	[90]
Ionic gelation	Sponge	Porous, decreased tensile strength	Antibacterial properties, improved healing	[91]
Solvent evaporation	Film	pH dependent swelling ration	No platform performance data presented	[92]
Ionic gelation and freeze-drying	Scaffold	Porous, high swelling ratio, Slow degradation	Antibacterial activity	[93]
Electrospinning	Scaffold	Micro-size porous structure	Aid cell attachment and proliferation	[94]
Electrospinning	Bilayer membrane	3-D porous structure	Antibacterial properties with maintained cell viability	[65]
Grafting	-	-	Improved water uptake	[60]
Solvent casting	Film	Enhanced tensile strength, decreased flexibility	High water uptake, aid wound healing	[95]
Solvent casting	Membrane	Porous, enhanced mechanical properties	Aid cell proliferation and maintains cell viability	[96]
Ionic gelation	Hydrogel	Small pore size porous structure, elastic and biodegradable	Cytocompatible, antibacterial properties, aid wound healing, decreased blood loss	[97]
Solvent casting	Film	Flexible, moderate drug release	Low cytotoxicity	[98]
Free radical polymerisation	Sponge	Porous and flexible	90% burst release in 4 h	[99]
Solvent droplet	Beads	-	Prolonged antibacterial activity	[100]
Ionic gelation	Sponge	Porous, high swelling ratio	Antibacterial, aid wound healing	[37]
Ionic gelation	Hydrogel	Porous and high swelling ratio	Aid cell growth	[101]
Padding and ionotropic gelation	Gauze and nanoparticles	Moderate water uptake	Poor antimicrobial properties	[102]
Electrospining	Scaffold	Nano size	Antibacterial, antioxidant and accelerated wound healing	[46]
Dissolution	Sponges	Porous thereby facilitating high fluid absorption	Cytocompatible and antibacterial activity	[103]
Solution casting	Membranes	Porous, high swelling ratio	Cytocompatible	[38]
Electrospinning	Nanofiber	Porous and High swelling ratio	Antimicrobial properties	[75]
Ionic gelation	Sponge	Porous, low tensile strength and high swelling ratio	Antibacterial properties with inflammation induction in cells	[104]
Ionic gelation	Gel	Porous structures, high drug loading, and swelling	-	[59]
Needle punching process	Gauze	Porous thereby facilitating high fluid absorption	Aid blood clotting, and blood absorption	[105]
Polymer coating	Membrane microfiber	Porous structure	Enhanced wound healing	[87]
Dissolution with wet-dry-spinning	Mats	Maintained thermal stability with improved tensile strength	Enhanced wound closure and cell attachment	[78]
Ionic gelation and droplet extrusion	Hydrogel	Lowered swelling ratios, stable mechanical properties (G′ and G″)	Effective antibacterial properties	[36]
Ionic gelation	Membranes	Higher tensile strength, acceptable fluid uptake, improved polymer dispersion and porosity	no antibacterial properties	[106]
Ionic gelation	Hydrogel sheets	High fluid uptake	Stimulated wound healing	[107]
Ionic gelation approach	Hydrogel	Optimum mechanical properties, porous, and high fluid uptake	Fast re-epithelialisation and formation of granulation tissues rate	[108]
Ionic gelation	Hydrogel	Optimum mechanical properties	Enhanced wound healing rate	[42]
Ionic gelation	Fibres	Optimum fluid uptake and mechanical properties	Cytocompatible, Improved wound healing and EGF expression	[48]
Ionic gelation	Nanofibers	Nano size and high viscosity	Enhanced wound healing rate	[83]

**Table 2:** Chitosan interactive compounds: polymers; bioactives; and crosslinkers.

Chitosan Interactive Compounds (Polymers, Lipids, and Proteins)	Interactive Bioactives	Crosslinkers	Ref.
Silk fibroin	-	-	[67]
Gelatine	Fe_3_O_4_	-	[68]
Gelatine	-	Glutaraldehyde	[71]
Alginate	AgNPs	CaCl_2_	[75]
Methoxy poly(ethylene glycol)	VEGF-PDGF-BB	Visible light irradiation and glycidyl methacrylate	[86]
Partially oxidised Bletilla striatapolysaccharide	AgNPs	Genipin	[109]
Collagen	-	Alginate	[90]
Collagen	-	Tannin acid	[91]
-	Ag–ZnO	-	[37]
Glutamic acid and Hyaluronic acid	Ag	-	[104]
Alginate	-	CaCl_2_	[59]
Collagen	TiO_2_	-	[60]
Polyethylene glycol	-	-	[87]
Collagen	-	Transglutaminase biocatalyst	[93]
-	TiO_2_	-	[96,110,111]
PVA and cyclodextrins	Ibuprofen	-	[38,112]
Polyacrylamide	-	Itaconic acid	[92]
-	-	Succinic anhydride	[88]
-	Silver sulfadiazine	-	[103]
Glucan	-	-	[78]
Gelatine	-	Glutaraldehyde	[58]
Gelatine	-	-	[44]
Alginate	-	CaC_l2_ as crosslinker, Pluronic F68 and Tween-80 as surfactants	[106]
Alginate	Fucoidan	CaCl_2_ and ethylene glycol diglycidyl ether	[107]
Alginate	Epidermal growth factor	CaCl_2_ and epidermal growth factor	[108]
Alginate	D-glucono-δ-lactone	-	[42]
Alginate and collagen	-	-	[48]
Alginate	Arginine	-	[83]
